# SIRT1: A promising therapeutic target for chronic pain

**DOI:** 10.1111/cns.13838

**Published:** 2022-04-09

**Authors:** Fan‐He Song, Dai‐Qiang Liu, Ya‐Qun Zhou, Wei Mei

**Affiliations:** ^1^ Anesthesiology Institute Tongji Hospital Tongji Medical College Huazhong University of Science and Technology Wuhan China; ^2^ Department of Anesthesiology and Pain Medicine Tongji Hospital Tongji Medical College Huazhong University of Science and Technology Wuhan China

**Keywords:** cancer‐induced bone pain, chronic pain, inflammatory pain, neuropathic pain, sirtuin 1

## Abstract

Chronic pain remains an unresolved problem. Current treatments have limited efficacy. Thus, novel therapeutic targets are urgently required for the development of more effective analgesics. An increasing number of studies have proved that sirtuin 1 (SIRT1) agonists can relieve chronic pain. In this review, we summarize recent progress in understanding the roles and mechanisms of SIRT1 in mediating chronic pain associated with peripheral nerve injury, chemotherapy‐induced peripheral neuropathy, spinal cord injury, bone cancer, and complete Freund's adjuvant injection. Emerging studies have indicated that SIRT1 activation may exert positive effects on chronic pain relief by regulating inflammation, oxidative stress, and mitochondrial dysfunction. Therefore, SIRT1 agonists may serve as potential therapeutic drugs for chronic pain.

## Graphical abstract

SIRT1 agonists can relieve chronic pain through regulating inflammation, oxidative stress, synaptic plasticity, and mitochondrial dysfunction. Activating SIRT1 becomes a promising treatment for chronic pain.

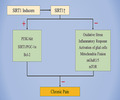

## 1 INTRODUCTION

The International Association for the Study of Pain (IASP) defines chronic pain as pain that persists for more than 3 months without biological value^1^ and persists past normal tissue healing. Chronic pain is a highly debilitating condition that influences all aspects of daily life in social and career‐related contexts.^2^ Opioids are powerful analgesics that are commonly used clinically. However, opioids can produce significant side effects, including respiratory depression, mental clouding, physical dependence, constipation, nausea, and vomiting.^3^, ^4^ The pharmacological tools currently available for treating chronic pain are severely inadequate or, as in the case of opioids, limited by serious side effects.^5^ Thus, efforts are needed to pursue research on innovative, nonopioid, pain‐relieving compounds.

Despite rapid advances over the past decades, the pathogenesis of chronic pain remains unclear.^6^, ^7^, ^8^ However, inflammation, oxidative stress, synaptic plasticity, and mitochondrial dysfunction are gradually being considered the primary causes of chronic pain.^9^, ^10^ Sirtuins are an evolutionarily highly conserved class of nicotinamide adenine dinucleotide (NAD+)‐dependent histone deacetylases that participate in many important cellular biological processes.^11^ Sirtuin 1 (SIRT1) is the best‐studied member of the sirtuin family because of its crucial role in many biological processes, including DNA repair and apoptosis, muscle and fat differentiation, neurogenesis, mitochondrial biogenesis, glucose and insulin homeostasis, hormone secretion, cell stress responses, and circadian rhythms.^12^, ^13^, ^14^, ^15^ SIRT1 is expressed in various tissues and cells in vivo, including the central nervous system, cardiomyocytes, hepatocytes, glomerular cells, and skeletal muscles.^12^ Under physiological conditions, SIRT1 is present in the nucleus and cytoplasm and acts mainly in the nucleus to deacetylate transcription factors such as peroxisome proliferator‐activated receptor‐γ coactivator‐1α (PGC‐1α), p53, forkhead box O, and nuclear factor‐kappa B (NF‐κB).^16^ Growing evidence has shown that SIRT1 plays an important role in reducing inflammation and oxidative stress and in improving mitochondrial function.^17^, ^18^, ^19^ Thus, SIRT1 may be a promising therapeutic target in the treatment of chronic pain.

Lower expression levels of SIRT1 have been observed in the spinal cord of various pathological pain models.^20^, ^21^, ^22^ Serum SIRT1 levels were negatively correlated with pain scores in patients with chronic pain. Zhang et al. found that the expression level of SIRT1 in the dorsal root ganglia (DRG) is related to pain vulnerability. SIRT1 interacts with the ClC‐3 channel and mediates ClC‐3 membrane trafficking and Cl‐currents in DRG neurons.^23^ SIRT1 deacetylates RelA/p65 to inhibit the nuclear translocation of NF‐κB, thus inhibiting inflammation.^24^ Intrathecal injection of a lentiviral vector overexpressing SIRT1 was shown to alleviate mechanical allodynia or thermal hyperalgesia.^25^ SIRT1 overexpression mediated by the lentiviral vector suppressed interleukin (IL)‐1 beta‐induced extracellular matrix degradation and cell apoptosis, alleviating the low back pain caused by intervertebral disk degeneration.^24^ Accumulating evidence has demonstrated that enhanced synaptic plasticity of nociceptive interneurons in the spinal dorsal horn is the basis of central sensitization in neuropathic pain.^26^, ^27^, ^28^ SIRT1 may influence nociceptive plasticity by enhancing translation in sensory neurons.^29^, ^30^, ^31^ Moreover, SIRT1 is involved in the development of nociceptor sensitization and central sensitization.^32^, ^33^, ^34^, ^35^ Previous studies have suggested a strong association between SIRT1 and chronic pain, indicating that targeting SIRT1 may lead to novel therapeutic interventions for the management of chronic pain. Moreover, SIRT1 activators have exhibited remarkable efficacy and safety against numerous diseases.^36^, ^37^ Here, we review the current evidence on the role of SIRT1 in the generation of chronic pain caused by peripheral nerve injury, chemotherapy‐induced peripheral neuropathy, spinal cord injury, bone cancer, and complete Freund's adjuvant (CFA) injection.

## 2 SIRT1 AND NEUROPATHIC PAIN

Chronic neuropathic pain is caused by a lesion or disease of the somatosensory nervous system. The pain may be spontaneous or evoked as an increased response to a painful stimulus (hyperalgesia) or a painful response to a normally nonpainful stimulus (allodynia). Chronic neuropathic pain can be divided into chronic peripheral neuropathic pain and chronic central neuropathic pain.^38^ Chronic peripheral neuropathic pain is commonly observed in trigeminal neuralgia, chronic neuropathic pain after peripheral nerve injury, and postherpetic neuralgia. Chronic central neuropathic pain is commonly observed in spinal cord injuries, multiple sclerosis, and brain injuries. Various animal models have been established to explore the mechanisms of neuropathic pain with different etiologies, including peripheral nerve injury, spinal cord injury (SCI), and chemotherapy‐induced peripheral neuropathy. Scientists have conducted numerous basic research studies using animal models to elucidate the mechanisms underlying neuropathic pain. Inflammatory responses, oxidative stress, immune system dysfunction, and epigenetic changes are considered to be involved in the pathogenesis of neuropathic pain.^39^, ^40^, ^41^, ^42^, ^43^, ^44^ SIRT1 has been reported to play a key role in neuropathic pain caused by peripheral nerve injury, SCI, and chemotherapy‐induced peripheral neuropathy.^45^, ^46^, ^47^, ^48^, ^49^

### 2.1 SIRT1 and peripheral nerve injury

Most studies have used animal models of peripheral nerve injury to explore the relationship between SIRT1 expression and neuropathic pain. Recent research has demonstrated that SIRT1 plays a critical role in the pathogenesis of neuropathic pain. Chronic constriction injury (CCI) was induced using the method described by Bennett and Xie.^50^ Yin et al. observed significantly lower paw withdrawal thresholds (PWT) and thermal withdrawal latency (TWL) in rats in the CCI group than in those in the sham group, verifying the successful development of mechanical allodynia and thermal hyperalgesia. Moreover, they noticed that SIRT1 levels were remarkably lower in the ipsilateral spinal cords of rats after CCI than those in sham‐operated rats.^51^ Given that SIRT1 consumes NAD and releases nicotinamide (NAM), they further investigated the effect of CCI surgery on NAD and NAM levels. In contrast to the sham group, the NAD content was lower and the NAM content was higher in CCI mice, which was consistent with the difference in SIRT1 levels.^52^ Additionally, compared with human bone marrow mesenchymal stem cell (hMSC)‐control implantation, intrathecal injection of hMSCs overexpressing SIRT1 (hMSCs‐SIRT1) exerted superior effects on alleviating neuropathic pain in CCI rats by reducing proinflammatory cytokine levels in the serum and spinal dorsal horn.^17^ These results indicate that SIRT1 may be involved in the development of neuropathic pain following CCI. Resveratrol (3,49,5‐trihydroxystilbene), a phytoalexin naturally present in plants, binds to SIRT1 at the N‐terminus, thus increasing SIRT1 activity.^53^ Intrathecal injection of 5 mL 90 mM resveratrol 1 h before CCI surgery^54^, ^55^ delayed the initiation of thermal hyperalgesia and mechanical allodynia.^51^ In addition, EX‐527 (6‐chloro‐2, 3, 4, 9‐tetrahydro‐1‐H‐carbazole‐1‐carboxamide), a SIRT1 inhibitor that is more potent and selective than other current SIRT1 inhibitors, can induce p53 acetylation and cell death by targeting SIRT1.^56^, ^57^ Shao et al. demonstrated that intrathecal injection of 5 mL EX‐527 (1.2 mM) 1 h before resveratrol administration effectively blocked the antinociceptive effect of 90 mM resveratrol.^52^ These results suggest that SIRT1 may be a major factor in the analgesic effects of resveratrol. However, the underlying molecular and cellular mechanisms have not been clearly investigated.

Adenosine 5′‐monophosphate (AMP)‐activated protein kinase (AMPK), is a ubiquitous kinase regulated by a variety of pharmacological entities.^58^ AMPK has been found to play a role in neuronal function, plasticity, and neurodegeneration as an “energy sensor.” Recently, Melemedjian et al. reported that AMPK activation could inhibit the development of neuropathic pain.^59^ Interestingly, SIRT1 is downstream of AMPK.^60^ Troxerutin, a derivative of the natural flavonoid rutin, relieved neuropathic pain in CCI rats.^61^ Gui et al. found that daily treatment with troxerutin after CCI significantly increased the expression and activity of SIRT1 and phosphorylation of AMPK in the spinal cord. However, EX‐527 decreased the levels of pAMPK and SIRT1 and reversed the effect of troxerutin on the pain thresholds of each group. This proved that troxerutin perturbs the development of neuropathic pain mainly through the activation of the AMPK/SIRT1 pathway.^61^ AMPK and SIRT1 have also been reported to inhibit the NF‐κB pathway.^62^, ^63^, ^64^, ^65^ NF‐κB, a member of the NF‐κB/Rel protein family, is a fast‐inducing transcription factor. In the acute phase of inflammation, mitochondria produce excess reactive oxygen species (ROS) that further activate NF‐κB to induce the expression of proinflammatory mediators, aggravating the inflammatory response and resulting in damage to the organism.^66^, ^67^ Excessive ROS‐mediated oxidative stress aggravates pain by activating the NF‐κB pathway.^68^, ^69^ SIRT1 inhibits the NF‐κB signaling pathway by deacetylating p65, a subunit of NF‐κB, thereby alleviating inflammatory responses and oxidative stress.^70^ A subsequent study found that persistent hyperalgesia and allodynia caused by CCI were associated with downregulation of SIRT1 in the spinal cord, which was reversed after intrathecal injection of the SIRT1 agonist, SRT1720. Injection of SRT1720 alleviates neuropathic pain by inhibiting the acetylation of NF‐κB and blocking the release of inflammatory factors, including tumor necrosis factor‐α (TNF‐α) and interleukin (IL)‐6.^71^ These data suggest that SIRT1 in the spinal cord plays an important role in neuropathic pain in rats.

There is emerging evidence that aberrant immune system responses contribute substantially to the generation of neuropathic pain.^72^ Immune cells respond to nerve injury by migrating into the nervous system at the site of injury, releasing mediators that affect intercellular signaling.^73^ Although the precise role of immune cells in neuropathic pain remains unclear, adoptive transfer of immune cells can increase pain by producing proinflammatory cytokines. In addition, the transfer of cells decreases pain sensitivity in nerve‐injured rats by producing anti‐inflammatory cytokines.^74^ In particular, the T cell response is considered a critical inducer of neuropathic pain.^75^, ^76^ Regulatory T cells (Tregs) are a subset of T cells, defined by the expression of CD4 and CD25 and the transcription factor forkhead box‐p3 (Foxp3), which is indispensable for the maintenance of self‐tolerance and immune homeostasis.^77^, ^78^ In animal models of peripheral nerve injury, the expansion of Tregs leads to a considerable reduction in pain hypersensitivity by limiting the immune responses of proinflammatory T cells. However, depletion of Tregs elevates pain hypersensitivity by inducing altered systemic concentrations of cytokines in mice.^72^, ^79^ SIRT1 directly colocalizes with Foxp3 and mediates its deacetylation and polyubiquitination, and Foxp3 is the master regulator of Treg differentiation.^80^, ^81^ In vitro data show that knockdown of SIRT1 in human CD4+ T cells induces Treg differentiation and increased *Foxp3* mRNA expression, indicating that a decrease in SIRT1 expression contributes to an increase in Treg cells in patients with neuropathic pain.^82^ MicroRNA (miR)‐124a and miR‐155 both regulate SIRT1 expression by directly targeting specific binding sites within the 3′ untranslated region (3′‐UTR) of SIRT1. Jens et al. found that patients with neuropathic pain had higher expression of miR‐124a than that in healthy volunteers, and miR‐155 inhibited SIRT1 expression, which enhanced CD4+ T cell differentiation towards Tregs.^82^ These studies indicate that SIRT1 may exert its effects on neuropathic pain by regulating Treg induction. Deciphering miRNA‐target interactions that influence inflammatory pathways in neuropathic pain may contribute to the discovery of new avenues for pain amelioration.

Previous studies have shown that SIRT1 protein, mRNA, and activity levels are lower in the spinal cord of diabetic neuropathic pain (DNP) rats.^83^ A well‐known SIRT1 activator, resveratrol, has been reported to ameliorate pain in diabetic neuropathy.^51^ It was also found that SRT1720 (an activator of SIRT1) suppresses thermal hyperalgesia and mechanical allodynia in DNP rats. Moreover, knockdown of spinal SIRT1 causes thermal hyperalgesia and mechanical allodynia in normal rats. These studies indicate that there might be an association between SIRT1 modulation and type 2 diabetes mellitus (T2DM)‐induced pain.^83^ SIRT1 may act as a promising molecular target for pain prevention and relief in patients with T2DM. A growing number of studies have demonstrated that alterations in gene expression due to epigenetic changes, such as anomalous histone acetylation in pain‐related genes in neurons, play a key role in the development and maintenance of neuropathic pain.^84^, ^85^, ^86^, ^87^ Histone acetylation is regulated by histone acetyltransferases and histone deacetylases, such as SIRT1.^88^ The induction of pain is dependent on the activation of certain ion channels in pain perception pathways. mGluR1 and mGluR5 are group I metabotropic glutamate receptors (mGluRs), which play vital roles in nociceptive processing by increasing the opening of cation channels, such as Ca^2+^, Na^+^, and K^+^ channels.^89^, ^90^ mGluR1 expression is regulated by histone 3 lysine 9 (H3K9) acetylation at the *Grm1* (encoding mGluR1) promoter region.^91^ Zhou et al. found that the expression of mGluR1/5 was higher in animals with DNP than in control rats, and the increased expression of mGluR1/5 was decreased by intrathecal injection of SRT1720.^20^ Interestingly, they also found that intrathecal injection of SRT1720 reduced pain‐induced H3 acetylation in the Grm1/5 promoter regions. These results indicate that SIRT1 attenuates DNP by inhibiting histone acetylation in the Grm1/5 promoter regions and subsequently inhibiting transcription.^20^ A recent study reported that SIRT1 also alleviated DNP by regulating synaptic plasticity of spinal dorsal horn neurons.^29^ In the spinal dorsal horn of DNP rats, decreased SIRT1 expression is associated with enhanced structural synaptic plasticity. Upregulation of spinal SIRT1 by the SIRT1 activator SRT1720 relieves pain behavior and inhibits enhanced structural synaptic plasticity in rats.^29^ These results demonstrate that SIRT1 plays an important role in rat DNP. Therefore, targeting SIRT1 may represent a novel therapeutic approach for neuropathic pain induced by T2DM.

### 2.2 SIRT1 and chemotherapy‐induced peripheral neuropathy

Chemotherapy‐induced peripheral neuropathy (CIPN) is a common consequence of several antineoplastic agents that can severely affect patients’ long‐term quality of life. However, the mechanisms underlying CIPN remain unclear.^92^, ^93^ Paclitaxel is a widely used chemotherapeutic drug, and one of its most common side effects is dose‐dependent painful neuropathy. Paclitaxel‐induced neuropathic pain (PINP) affects the quality of patients’ lives and still lacks an effective treatment.^94^, ^95^ Several studies have demonstrated that spinal neuroinflammation is linked to the formation of PINP. Li et al. reported that NF‐κB (p65)‐dependent histone 4 (H4) acetylation elevates the production of proinflammatory factors such as CX3CL1.^96^ Thus, H4 acetylation may be a potential target in the treatment of PINP. SIRT1 activation has been reported to decrease H4 acetylation (particularly at H4‐K16) and relieve neuropathic pain.^97^ Rats were intraperitoneally injected with paclitaxel (8 mg/kg) for 3 days (D1, D4, and D7) to establish PINP models. The cumulative dose of paclitaxel was 24 mg/kg.^98^ Gui et al. found that paclitaxel treatment inhibited SIRT1 activity in the spinal cord.^99^ They also found that icariin treatment considerably alleviated paclitaxel‐induced neuropathic pain and reversed paclitaxel‐induced SIRT1 downregulation and H4‐K16 acetylation. Furthermore, EX‐527 eliminated the analgesic effect of icariin while blocking icariin‐induced SIRT1 upregulation and H4‐K16 deacetylation. This indicates that SIRT1 plays a crucial role in icariin‐induced effects. Icariin suppresses paclitaxel‐induced neuroinflammation and mechanical allodynia in a SIRT1‐dependent manner. Thus, icariin could be a potential agent for the treatment of PINP.

Vincristine (Vin) is another well‐known antitumor agent that frequently induces neuropathic pain and decreases the quality of life of patients. Xie et al. found that SIRT1 activity and expression were significantly lower in the sciatic nerve, spinal cord, and DRG of rats in the Vin group than in the naive group.^100^ The rhizome of *Gastrodia elata* Blume (*G*. *elata*) is used as a traditional herbal medicine. Polysaccharides (GBP) extracted from *Gastrodia elata* Blume have been demonstrated to possess anti‐inflammatory and neuroprotective effects in vivo.^101^, ^102^ GBP relieved Vin‐induced neuropathic pain by decreasing proinflammatory cytokine levels and activating SIRT1 expression in the spinal cord and DRG. Xie et al. demonstrated that GBP treatment decreased NF‐κB levels in the spinal cord and DRG, which suppressed IL‐6, IL‐8, IL‐1β, and TNF‐α release.^100^ Thus, GBP may be a promising therapeutic agent for the management and alleviation of neuropathic pain.

### 2.3 SIRT1 and spinal cord injury

Spinal cord injury (SCI) results from neurological damage in the spinal cord and leads to serious impairment of sensorimotor function, along with other effects^103^ such as paraplegia and tetraplegia.^104^, ^105^ Patients with SCI experience pain.^106^ Substantial research has explored the pathophysiological changes that occur following SCI.^107^ However, how to most effectively repair the spinal cord after damage remains unclear. The systemic inflammatory response is considered a key factor leading to the development of SCI‐induced immunological dysfunction.^108^, ^109^ However, the role of the inflammatory response in SCI remains unclear. Therefore, it is important to further clarify the molecular mechanisms underlying SCI to develop novel therapeutic strategies. Yu et al. conducted a study to investigate the role of SIRT1 in SCI pain by using a clinically relevant rat contusion model.^21^ Hematoxylin and eosin staining showed that the inflammatory response was considerably greater in SCI tissues compared to that in the control group. Levels of SIRT1 protein and mRNA in SCI tissues were significantly lower compared to those in the control group.^21^ Neuronal apoptosis is suppressed following nervous system injury.^110^ However, several proteins and nuclear transcription factors are specifically expressed in a delayed manner, and p53 is one of the key molecules in the cell apoptosis pathway. Upregulated p53 expression can directly induce cell apoptosis.^110^ Yu et al. found that SIRT1 may inhibit apoptosis of SCI in vivo and in vitro through the p53 signaling pathway, whereas miR‐494 may suppress SIRT1 and induce apoptosis.^21^ In a subsequent study, Chen et al. reported that miR‑138‑5p could modulate the PTEN/AKT signaling pathway via SIRT1, thus regulating the inflammatory response and cell apoptosis in SCI models, contributing to the development of SCI.^111^ These findings suggest that miRNAs may serve as novel therapeutic targets in the treatment of SCI via SIRT1.

## 3 SIRT1 AND CANCER‐INDUCED BONE PAIN

Bone cancer pain (BCP) is pain induced by primary bone cancer or tumor metastasis. Bone metastasis is more common in patients with breast, prostate, kidney, and lung cancer.^112^ Although a variety of factors are linked to BCP, the specific cellular and molecular mechanisms underlying its pathogenesis remain unclear, and effective clinical approaches are urgently needed for its treatment.

SIRT1 plays a critical role in BCP.^113^ SIRT1 expression and activity are lower in rats with BCP. However, SRT1720 (an activator of SIRT1) treatment reverses pain behavior in BCP rats and upregulates SIRT1.^114^, ^115^ Neurons are metabolically active cells with high energy demands and are particularly dependent on mitochondrial function.^116^ Dynamin‐related protein 1 (Drp1), a cytosolic guanosine‐5′‐triphosphatase, migrates between the cytosol and mitochondrial network and binds to the mitochondrial outer membrane, driving mitochondrial fission.^117^ Upregulation of Drp1 causes mitochondrial fragmentation and mitochondrial membrane potential (MMP) reduction, which is an initial and irreversible step towards apoptosis.^118^ In the spinal cord of BCP rats, the expression of the mitochondrial fission‐related protein, Drp1, is increased. Injection of SRT1720 reduces Drp1 expression to normal levels in rats with BCP. Moreover, decreased expression of Drp1 is observed in an SRT1720 dose‐dependent manner.^114^ Ample evidence suggests that PGC‐1α is activated by deacetylation via SIRT1 activation. PGC‐1α subsequently promotes mitochondrial gene transcription and mitochondrial biogenesis.^119^ The SIRT1/PGC‐1α pathway regulates Drp1 expression and mitochondrial fission. Overexpression of PGC‐1α reduces Drp1 expression and promotes Drp1‐mediated mitochondrial biogenesis. Mutation of PGC‐1α reduces mitochondrial function and oxidative capacity.^120^ These findings indicate that SRT1720 reverses pain behavior by activating the SIRT1/PGC‐1α/Drp1 pathway, which consequently inhibits mitochondrial fission‐associated apoptosis. In a further study, Yang et al. proved that SIRT1 activation in the spinal cord caused by SRT1720 also reversed BCP in mice by inhibiting mGluR1/5.^121^ Moreover, it has been reported that the activation of the cyclic adenosine monophosphate response element‐binding (CREB) protein also plays a critical role in BCP.^122^, ^123^ SIRT1 regulates the balance between glucose and lipid metabolism through CREB deacetylation.^124^, ^125^ A recent study suggested that intrathecal administration of SRT1720 reversed BCP, likely by inhibiting the CREB/ CREB‐regulated transcription coactivator 1 (CRTC1) signaling pathway. These findings suggest that SIRT1 is a potential target for treating BCP.

## 4 SIRT1 AND INFLAMMATORY PAIN

Inflammatory pain is a common clinical symptom of inflammatory diseases. It is characterized by hyperalgesia due to sensitization of primary nociceptive neurons.^126^, ^127^ Pain hypersensitivity is caused by the release of inflammatory mediators from immune and non‐neuronal cells in the periphery. Proinflammatory cytokines (PICs), such as IL‐1β, IL‐6, and TNF‐α, play essential roles in pain sensitization.

In recent years, SIRT1 has been shown to exert anti‐inflammatory and antioxidative effects and to alleviate cell injury. The SIRT1‐related anti‐inflammatory mechanism is complex and involves numerous pathways, including the mitogen‐activated protein kinase and NF‐κB pathways.^128^ SIRT1 markedly reduces the activity of the NF‐κB pathway and decreases the production of inflammatory factors.^129^ In addition, it has been reported that SIRT1 also inhibits inflammatory response‐induced injury in proto‐cortical neurons. A possible mechanism underlying these effects is that SIRT1 acts on the NF‐κB subunit through deacetylation. In particular, SIRT1 reduces the binding of NF‐κB to intranuclear inflammatory genes and the production of inflammatory factors, including TNF‐α and IL‐1β. Furthermore, it has been suggested that the cyclooxygenase pathway may also be involved.^128^

Previous reports have demonstrated that miRNAs are key regulators of physiological and pathological processes underlying pain. This suggests that specific miRNAs may be useful as novel molecular targets for the prevention and relief of chronic pain.^130^, ^131^, ^132^ However, the direct association between spinal miR‐34a expression and inflammatory pain remains unclear. Recently, Chen et al.^133^ found that miR‐34a levels were significantly increased in the spinal cord of CFA‐administered mice, consistent with their pain behavior. Overexpression of miR‐34a induces pain, mechanical allodynia, and thermal hyperalgesia in naive mice. Furthermore, intrathecal injection of miR‐34a antagomir in CFA‐treated mice alleviates the nociceptive response induced by CFA injection. These data suggest that miR‐34a is an endogenous initiator of pain, and exogenous supplementation with synthetic miR‐34a antagomir exerts an analgesic effect on CFA‐induced inflammatory pain. In contrast to the inflammatory pain induced by increased miR‐34a expression, SIRT1 expression was found to be decreased in the spinal cord of mice administered CFA. Subsequent evidence indicated that miR‐34a participates in the suppression of SIRT1 expression by binding to the 3′‐UTR of SIRT1.^134^, ^135^, ^136^ Intrathecal injection of miR‐34a antagomir relieves CFA‐induced mechanical allodynia and thermal hyperalgesia. Interestingly, it also reduces inflammatory pain‐induced SIRT1 downregulation. Furthermore, by analyzing whether miR‐34a mediates pain modulation by negatively regulating SIRT1 expression, Chen et al. revealed that inhibition of SIRT1 by EX‐527 effectively inhibits the antinociceptive effect of the miR‐34a antagomir. These in vivo data indicate that miR‐34a relieves inflammatory pain by inhibiting SIRT1 expression in the spinal cord. Thus, miR‐34a‐SIRT1 signaling may serve as a novel therapeutic target for inflammatory pain.

## 5 CONCLUDING REMARKS AND FUTURE PERSPECTIVE

**FIGURE 1:**
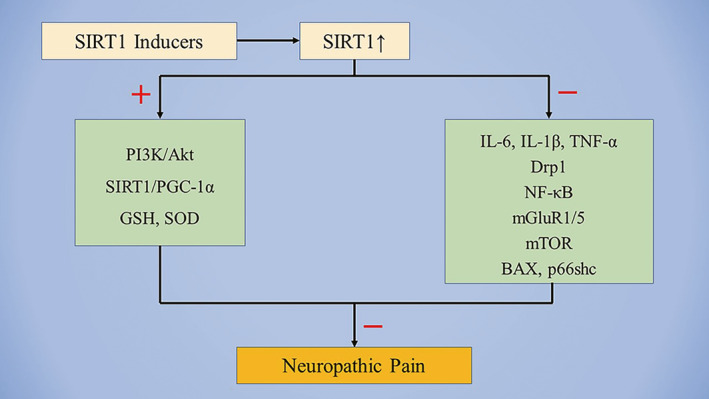
Schematic representation of the downstream mechanism of SIRT1 in the neuropathic pain. Abbreviations: Akt, protein kinase B; BAX, B‐cell lymphoma2‐associated X protein; Drp1, dynamin related protein 1; GSH, glutathione; IL, interleukin; mGluR, metabotropic glutamate receptor; NF‐κB, nuclear factor‐kappa B; PGC‐1α, peroxisome proliferator‐activated receptor‐γ coactivator‐1α; PI3K, phosphatidylinositol 3‐kinase; SOD, superoxide dismutase; TNF‐α, tumor necrosis factor‐α; +, upregulated; ‐, downregulated

**FIGURE 2:**
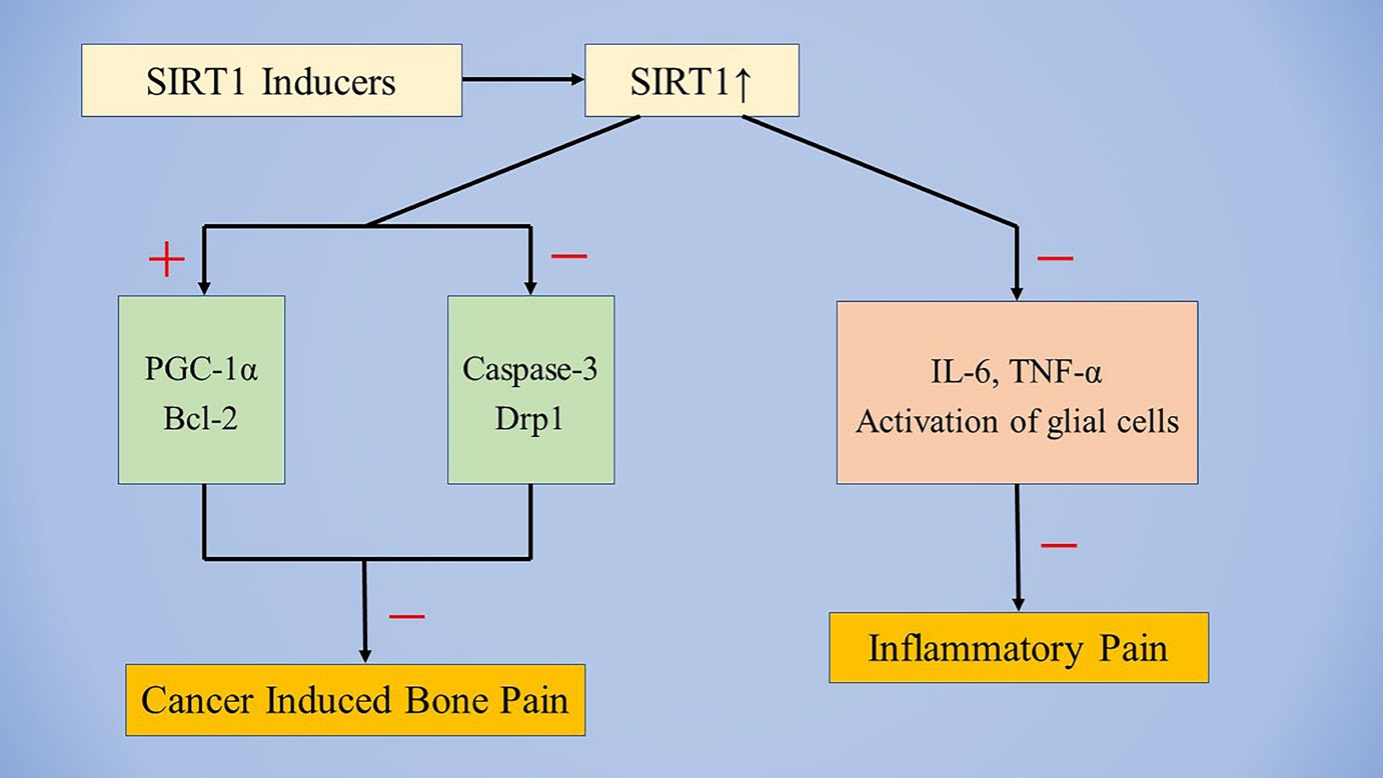
Schematic representation of the possible upstream mechanism of SIRT1 in the processing of cancer induced bone pain and inflammatory pain. Abbreviations: Bcl‐2, B‐cell lymphoma2; Drp1, dynamin related protein 1; IL, interleukin; PGC‐1α, peroxisome proliferator‐activated receptor‐γ coactivator‐1α; TNF‐α, tumor necrosis factor‐α; +, upregulated; ‐, downregulated

Although great progress has been made in pain research over the past decade, there has been little translation of preclinical results into clinical practice. Currently, very few novel therapeutic options have been provided to patients, and the side effects of traditional drugs are difficult to ignore. Therefore, patients are frequently undertreated, and more effective analgesic drugs are urgently needed. Previous studies have shown that SIRT1 plays a critical role in the pathogenesis of neuropathic pain, BCP, and inflammatory pain. Decreased expression SIRT1 levels have been observed in the spinal cord of various pathological pain models. Notably, treatment with SIRT1 activators considerably attenuated mechanical allodynia and thermal hyperalgesia caused by pathological pain (Table 1). While reviewing the current evidence, we have discussed the relationship between SIRT1 and chronic pain (Figures 1 and 2). SIRT1 overexpression may be a novel and beneficial therapeutic tool for chronic pain management. However, these findings raise additional questions.

**TABLE 1:** Summary of therapeutic potential of SIRT1 inducers in chronic pain

Compound	Model	Treatment strategy	Effects	Mechanisms	References
Resveratrol	CCI‐induced neuropathic pain mice	Resveratrol (300 μg/day, i.t.) was administered from day 4 to 7 after CCI	PWT↑ TWL↑	SIRT1↑ Acetyl‐histone H3↓	^51^
	LPS‐induced inflammation pain mice	Resveratrol (10 and 20 mg/kg, i.p.) was administered 30 min prior to administration of LPS	Number of writhes↓ Tail‐flick latency↑	SIRT1↑ IL−6↓, TNF‐α↓ Activation of glial cells in the dorsal horns of the spinal cord↓	^143^
	Paclitaxel‐induced neuropathic pain rats	Resveratrol (40 mg/kg, i.p.) was administered on seven alternate days (days 2, 4, 6, 8, 10, 12, and 14) after the first injection of paclitaxel	PWT↑ TWL↑	SIRT1/PGC1α↑ PI3K/Akt↑	^144^
	Bone cancer pain mice	Acute resveratrol (100 mg/kg, i.p.) was administered 14 days after the injection of cancer cells, or chronic resveratrol (100 mg/kg i.p.) was administered from day 14 to 20 after the injection of cancer cells	Functionality of the hind limb↑	SIRT1↑	^115^
Troxerutin	CCI‐induced neuropathic pain mice	Troxerutin (150 mg/kg, i.g.) was administered for 7 days after CCI	PWT↑ TWL↑	AMPK/SIRT1↑ IL−1β, TNF‐α, INF‐γ↓ Activation of microglia↓ NF‐κB (p65)↓	^61^
Carnosic acid	CCI‐induced neuropathic pain mice	Carnosic acid (25 and 100 μg, i.t.) was administered from day 3 to 6 after surgery	PWT↑ TWL↑	SIRT1↑ P66shc↓	^145^
SRT1720	T2DM‐induced neuropathic pain rats	SRT1720 (0.08 µg, i.t.) was administered 4 days after successful induction of diabetes	PWT↑ TWL↑	SIRT1↑ Fos protein↓ mGluR1/5↓	^20^
	Bone cancer pain rats	SRT1720 (0.5 mg/kg, i.t.) was administered 12 days after the injection of cancer cells	PWT↑	SIRT1↑ Drp1↓ Bcl−2↑ Caspase−3↓ PGC−1α↑	^114^
	CCI‐induced neuropathic pain mice	SRT1720 (0.05 µg, c.i.) was administered 12 days after CCI	TWL↑	SIRT1↑ Acetyl‐histone H3↓ Fos protein↓	^146^
Icariin	Paclitaxel‐induced neuropathic pain rats	Icariin (100 mg/kg, i.g.) was administered from day 8 to 15 after the first injection of paclitaxel	PWT↑	SIRT1↑ TNF‐α, IL−1β, IL−6↓ NF‐κB(p65)↓	^99^
Formononetin	T2DM‐induced neuropathic pain rats	Formononetin (20 and 40 mg/kg/day, i.g.) was administered for 16 weeks after confirmation of type 2 diabetes	PWT↑ MWT↑	SIRT1↑ GSH, SOD↑ MDA↓	^83^
Lycopene	Burn injury pain rats	Lycopene (40 nmol and 60 nmol, i.t.) was administered before behavior tests of BIP models	PWT↑	SIRT1↑ mTOR↓ GFAP, Iba−1↓	^147^
Astragaloside IV	Diabetic peripheral neuropathy rats	Astragaloside IV (60 mg/kg, i.g.) was administered every day for 12 weeks after the successful induction of diabetes	PWT↑	SIRT1↑ Drp1↓, p53↓ MDA↓, GSH↑ Caspase−3↓ BAX↓, BCL−2↑	^148^

Abbreviations: ↑, upregulated; ↓, downregulated; Akt, protein kinase B; AMPK, adenosine 5′‐monophosphate (AMP)‐activated protein kinase; BAX, BCL‐2‐associated X protein;Bcl‐2, B‐cell lymphoma‐2; BIP, burn injury pain; c.i., controlled infusion; CCI, chronic constriction injury; Drp1, dynamin‐related protein 1; GFAP, glial fibrillary acidic protein; GSH, glutathione; i.g., intragastric; i.p., intraperitoneal; i.t., intrathecal; Iba‐1, ionized calcium‐binding adaptor molecule 1; IL, interleukin; INF‐γ, interferon‐γ; LPS, lipopolysaccharide; MDA, malondialdehyde; mGluR, metabotropic glutamate receptor; NF‐κB, nuclear factor‐kappa B; PGC‐1α, peroxisome proliferator‐activated receptor coactivator‐1α; PI3K, phosphatidylinositol 3‐kinase; PWT, paw withdrawal thresholds; SOD, superoxide dismutase; T2DM, type 2 diabetes; TNF‐α, tumor necrosis factor α; TWL, thermal withdrawal latency.

First, whether SIRT1 activators exert similar analgesic effects on other types of chronic pain (e.g., cancer and osteoarthritis pain) requires further investigation. Each type of chronic pain has unique characteristics. However, they may have the same pathogenesis. Therefore, it is important to reveal the beneficial effects of SIRT1 inducers in other types of chronic pain.

Second, current studies have mostly focused on the analgesic effects of SIRT1 inducers rather than the underlying mechanisms. Therefore, the detailed mechanisms underlying the analgesic effects of SIRT1 inducers and the mechanisms by which so‐called SIRT1 inducers activate SIRT1 need to be elucidated. In addition, none of the SIRT1 activators mentioned in this review is a specific activator of SIRT1. Therefore, specific SIRT1 activators or transgenic rat models might be better for elucidating the role of SIRT1 in chronic pain. Despite the inspiring therapeutic efficacy of SIRT1 inducers against chronic pain in preclinical studies, no relevant clinical trials are available. Therefore, further studies are warranted.

Finally, current studies have primarily focused on the relationship between SIRT1 and chronic pain. However, other sirtuins (e.g., SIRT2, SIRT3, and SIRT6) are also involved in the mechanisms of chronic pain. For example, Zhang et al. found that SIRT2 overexpression inhibits the expression of TNF‐α, IL‐1β, and IL‐6 in the dorsal root ganglion of CCI rats, alleviating neuropathic pain associated with neuroinflammation.^137^ A study demonstrated that targeting SIRT3 to improve mitochondrial redox homeostasis might represent a potential therapeutic strategy for low back pain caused by IVD degeneration.^138^ It has been reported that SIRT3 improves the ability of mitochondria to reduce ROS levels and protect against oxidative stress by regulating the activity of key antioxidant enzymes, such as manganese superoxide dismutase.^139^, ^140^, ^141^ In lipopolysaccharide‐induced pulpitis, Hu et al. demonstrated that overexpression of SIRT6 led to a significant reduction in proinflammatory cytokines (IL‐6, IL‐1 β, and TNF‐α) and inactivation of the NF‐κB pathway, thereby attenuating pain caused by pulpitis.^142^ Therefore, the above evidence indicates that other sirtuins may act as potential targets for the prevention and treatment of chronic pain. Large‐scale, multicenter, prospective clinical trials are needed. Given the analgesic effects of sirtuin activators, efforts to discover more sirtuin activators would have a large impact on clinical and public health.

## CONFLICT OF INTEREST

The authors declare that there are no conflicts of interest.

## Data Availability

All data included in this study are available upon request by contact with the corresponding author.
